# A Premature Rise of Luteinizing Hormone Is Associated With a Reduced Cumulative Live Birth Rate in Patients ≥37 Years Old Undergoing GnRH Antagonist *In Vitro* Fertilization Cycles

**DOI:** 10.3389/fendo.2021.722655

**Published:** 2021-12-02

**Authors:** Fumei Gao, Yanbin Wang, Dan Wu, Min Fu, Qiuxiang Zhang, Yumeng Ren, Zexi Yang, Huan Shen, Hongjing Han

**Affiliations:** Reproductive Center of Peking University Peoples’ Hospital, Peking University, Beijing, China

**Keywords:** GnRH antagonist, premature rise of LH, IVF, cumulative live-birth rate, advanced aged patients

## Abstract

This is a retrospective cohort study included 1021 patients underwent a flexible GnRH antagonist IVF protocol from January 2017 to December 2017 to explore the effect of a premature rise in luteinizing hormone (LH) level on the cumulative live birth rate. All patients included received the first ovarian stimulation and finished a follow-up for 3 years. A premature rise in LH was defined as an LH level >10 IU/L or >50% rise from baseline during ovarian stimulation. The cumulative live birth rate was calculated as the number of women who achieved a live birth divided by the total number of women who had either delivered a baby or had used up all their embryos received from the first stimulated cycle. In the advanced patients (≥37 years), the cumulative live birth rate was reduced in patients with a premature rise of LH (β: 0.20; 95% CI: 0.05–0.88; *p*=0.03), compared to patients (≥37 years) without the premature LH rise. The incidence of premature LH rise is associated with decreased rates of cumulative live birth rate in patients of advanced age (≥37 years) and aggravated the reduced potential of embryos produced by the advanced age, not the number of embryos.

## Introduction

Over the last few years, gonadotropin-releasing hormone (GnRH) antagonists have been widely used for controlled ovarian stimulation (COS) in assisted reproductive technology (ART) (1). GnRH antagonists have several advantages over GnRH agonists, including a shorter treatment time, the reduced dosage of gonadotropic hormones per cycle, improved patient acceptance, and a significantly lower incidence of ovarian hyperstimulation syndrome (OHSS), particularly severe hyperstimulation (2–4). GnRH antagonists can inhibit a premature rise of luteinizing hormone (LH) during COS by competitively binding to GnRH receptors after immediate pituitary suppression (5). However, it has been reported that some patients still experience a premature rise of LH after treatment with GnRH antagonists (6). The clinical significance of such a rise remains unknown. Several studies have shown that a premature rise in LH level may result in a reduced ongoing pregnancy rate (7), whereas others indicate that a transient rise in LH is not associated with a decline in pregnancy rates (8, 9). However, it is worth noting that the patients involved in these previous studies underwent only fresh embryo transfer and did not consider the transfer of frozen embryos. Thus, the true effect of a premature rise in LH on embryos cannot be evaluated comprehensively. Moreover, a comparison of the cumulative pregnancy outcome after both fresh and frozen embryo transfers in cycles where a premature rise in LH level has occurred may help us to understand the effect of this premature LH rise on embryos and pregnancy outcome more directly. The aim of the present study was to identify the effect of a premature LH rise on cumulative live birth rate during IVF cycles involving a GnRH antagonist protocol.

## Materials and Methods

### Study Participants

This was a retrospective cohort study designed to examine the effect of a premature rise in LH on the cumulative live birth rate of patients who underwent a flexible GnRH antagonist protocol during ART. All GnRH-antagonist cycles performed at the Reproductive Center of Peking University Peoples’ Hospital from 1^st^ January 2017 to 31^st^ December 2017 were assessed.

All women between 18 and 45 years old undergoing their first ovarian stimulation cycle, in a GnRH antagonist protocol, were considered eligible patients and they were followed up for 3 years period. Each patient was included only once in the analysis. The exclusion criteria were the use of a protocol other than a GnRH antagonist protocol. The study was approved by the institutional review boards.

### Protocol for Controlled Ovarian Stimulation and Embryo Transfer

The patients did not receive oral contraceptive pill before the IVF cycle. Ovarian stimulation began on day 2 of the menstrual cycle with recombinant FSH (Gonal-F; Merck Serono, Coinsins, Switzerland) 150–450 IU daily with or without 75–300 IU of hMG (Livzon, Shanghai, China). Human menopausal gonadotropin (hMG) was used in patients when a poor response was anticipated because of advanced age, low antral follicle count, basal FSH >10 IU/L, or AMH <1.1 ng/mL. The starting dose of Gn (FSH/hMG) was based on the patient’s age, weight, height, antral follicle count (AFC), and hormonal profile. Doses were adjusted according to serum E2 and ovarian response which was evaluated by transvaginal ultrasound. The administration of GnRH-antagonist (0.25 mg daily of Ganirelix or Cetrotide, 10:00 am) was initiated based on a flexible protocol, generally when the lead follicle was 13 to 14 mm in diameter, and was continued until the day of hCG administration. Individuals experiencing a premature rise in LH were administered with an additional dose of GnRH antagonist on the day of the rise, with continued daily doses of 0.25 mg thereafter. When three follicles reached a mean diameter of 17 mm, or two follicles reached a mean diameter of 18 mm, final oocyte maturation was triggered by 250 mg of recombinant hCG (Ovidrel; Merck Serono) alone, which was equivalent to ~6,500 IU hCG according to manufacturer data, or by 0.2 mg of triptorelin (Ferring International Center, Saint-Prex, Switzerland) plus 2000 IU of hCG (Livzon, Zhuhai, China). Oocyte retrieval was performed by transvaginal ultrasonography 35–37 hours later. Intracytoplasmic sperm injection (ICSI) was only performed for patients experiencing severe male factor infertility. Fertilization was assessed 16–18 hours after insemination for the appearance of two distinct pronuclei and two polar bodies. The zygotes were cultured in a cleavage medium (Cook Medical). Embryonic development was assessed daily. Fresh embryo transfer was performed at day 3 after oocyte retrieval and supernumerary embryos of good quality were cryopreserved (vitrification protocol). Day 3 embryos of high quality were defined if they met the following criteria: 7 to 9 cells stage at day three after fertilization, fragmentation is less than 10% and homogenous blastomeres according to the ASEBIR consensus (10). If it does not meet the criterion of excellent quality embryos, embryos were cultured to the blastocyst stage in blastocyst medium (Cook Medical). Blastocyst quality scoring was performed on day 5 according to Gardner’s criteria (11). The score depended on blastocyst expansion, inner cell mass development, and trophectoderm appearance. Inner cell mass and trophectoderm scoring was performed, and according to their morphologic appearance blastocysts were graded as top quality (grade 1) (AA), good quality (grade 2) (AB and BA), average quality (grade 3) (AC, CA, BB), and poor quality (grade 4) (BC, CB, CC). Blastocysts with a score>3BB were vitrified either on day 5 or day 6 and also were defined as high quality blastocysts. All embryos would be frozen if a patient had issues related to a thin endometrial lining (<7 mm), intrauterine fluid, hydrosalpinx, elevated progesterone level (>1.5 ng/mL) on the day of HCG administration, or had a high risk of ovarian hyperstimulation.

During fresh embryo transfer cycles, embryos were transferred on day 3 (D3) after fertilization. In frozen-thawed embryo transfer cycles, embryos were transferred in natural cycles or in hormonal replacement cycles. Patients who failed ovulate were given estradiol valerate (Progynova, Bayer, German) from D3 of the menstrual cycle (3 mg twice daily orally). Progesterone (intramuscular progesterone at a daily dose of 60 mg) was administered when the thickness of the endometrium reached ≥8 mm. The number of transferred embryos varied from one to two, depending on embryo quality and patient age.

### Definition of a Premature LH Rise

A premature rise in LH was defined as an LH level >10 IU/L or >50% rise from baseline during ovarian stimulation, including the day of the trigger. LH level was determined at 10:00–12:00 am every two days from the beginning of the ovarian stimulation.

### Primary and Secondary Outcomes

The primary outcome measure in this study was cumulative live birth rate. The cumulative live birth rate was calculated as the number of women who achieved a live birth (>28 weeks of gestation) in the fresh or in the subsequent frozen-thawed cycles divided by the total number of women who had either delivered a baby or had used up all their embryos received from the first stimulated cycle.

Secondary outcome measures included implantation rate, clinical pregnancy rate and ongoing pregnancy rate. The implantation rate was calculated by dividing the number of gestational sacs by the number of embryos transferred. Clinical pregnancy was defined as a visible gestational sac and fetal cardiac activity on transvaginal ultrasonography. The ongoing pregnancy rate was defined as a pregnancy beyond 12 weeks gestation.

### Data Analysis

Cycle characteristics and secondary outcome measures were compared between the premature LH rise group and the non-LH rise group using the Student’s t-test and chi- squared measure of association for continuous and categorical variables, respectively. Logistic regression was used to determine whether the premature rise in LH was significantly associated with cumulative live birth rate. The logistic regression model was adjusted for age, etiology of infertility, basal FSH, basal LH, antral follicular count, duration of infertility, and body mass index (BMI).

## Results

A total of 1205 patients were treated with a flexible GnRH antagonist protocol between January 2017 and December 2017. Of these patients, 1021 patients underwent embryo transfer until either they achieved pregnancy or had used up all of the frozen embryos acquired from the first stimulated cycle. Of the 1021 patients, 82 patients (8.03%) experienced a premature rise in LH. Age, body mass index (BMI), basal FSH, antral follicle count (AFC), and the duration of infertility were similar when compared between women with and without a premature rise in LH (**Table 1**). There were no significant differences between the groups with or without a premature rise in LH rise in terms of the number of oocytes retrieved, the number of metaphase II oocytes, two pronuclei (2PN) embryos, available embryos, high-quality embryos, and endometrial thickness (**Table 1**). Notably, women with a premature rise in LH had significantly higher LH levels (5.43 ± 2.83 *vs* 4.26 ± 2.37, *p*=0.00) on the day of hCG administration compared with women without a premature rise in LH (**Table 1**).

**Table 1:** A comparison of baseline characteristics between cycles with and without a premature rise in LH level.

	No LH rise (n = 939)	LH rise (n = 82)	*p*
Age (y)	33.68 ± 4.51	33.88 ± 4.82	0.71
BMI	22.64 ± 3.87	22.20 ± 3.56	0.32
Duration of infertility (y)	3.65 ± 3.05	3.73 ± 2.95	0.81
Diagnosis			0.48
Pelvic or tubal factor	271 (28.86%)	21 (25.61%)	
DOR	62 (6.60%)	7 (8.54%)	
Endometriosis	57 (6.07%)	4 (4.88%)	
Ovulatory dysfunction/PCOS	55 (5.86%)	9 (10.98%)	
Male factor	198 (21.09%)	13 (15.85%)	
Both factor	229 (24.39%)	23 (28.05%)	
Unexplained	67 (7.14%)	5 (6.10%)	
Day 3-FSH (IU/L)	8.12 ± 3.81	8.12 ± 3.39	0.99
Day 3-LH (IU/L)	4.26 ± 2.37	5.43 ± 2.83	0.00^a^
Day-3 E2 (pg/ml)	41.74 ± 19.89	39.26 ± 20.72	0.46
Day-3 P (ng/ml)	0.36 ± 0.86	0.42 ± 0.67	0.72
Antral follicle count	10.24 ± 5.39	9.89 ± 6.59	0.59
Days of gonadotropin stimulation	9.55 ± 1.82	9.91 ± 1.85	0.09
Total gonadotropin dose	2164.95 ± 689.06	2162.50 ± 696.91	0.96
Progesterone on HCG trigger	1.21 ± 0.81	1.50 ± 1.34	0.06
LH at HCG trigger (IU/L)	3.12 ± 3.13	6.55 ± 5.83	0.00^a^
Endometrial thickness on HCG trigger (mm)	9.24 ± 2.59	9.12 ± 2.70	0.69
No of oocytes retrieved	11.19 ± 6.72	10.39 ± 6.24	0.30
Fertilization rate (%)	85.54 ± 19.18	85.53 ± 22.12	0.99
No. of MII oocytes	8.36 ± 5.47	7.63 ± 4.90	0.26
No. of 2PN	7.31 ± 4.72	8.12 ± 11.29	0.20
No. of available embryos	2.40 ± 1.09	2.29 ± 0.94	0.39
No. of high-quality embryos	1.19 ± 1.22	1.25 ± 1.21	0.65

BMI, body mass index; E2, estradiol; FSH, follicle-stimulating hormone; hCG, human chorionic gonadotropin; LH, luteinizing hormone; PCOS, polycystic ovary syndrome; DOR, diminished ovarian response.

a indicates a statistically significant p value (<0.05) via a two-sample t-test or chi-squared test for continuous and categorical variables, respectively.

Next, we investigated the influence of a premature rise in LH on pregnancy outcomes. Of all the 1021 patients, 269 patients underwent the transfer of fresh embryos while 752 patients underwent frozen embryo transfers (FET). There were 640 patients received one embryo transfer cycle and 296 patients received two embryo transfer cycles. Only 85 patients received more than three embryo transfer cycles. There was no difference between the number of embryos transferred, implantation rate, clinical pregnancy rate, ongoing pregnancy rate between the patients with and without a premature LH rise. In the present study, there are 264 patients ≥37 years old, among these 22 patients suffering from a premature LH surge and 242 patients without a LH rise. Though the cumulative live birth rate (51.76% *vs* 49.38%) were not significantly different between the two groups (**Table 2**), the hierarchical analysis based on female age showed that in the group of patients aged ≥37 years, a significantly reduced cumulative live birth (β: 0.20; 95% CI: 0.05–0.88; *p*=0.03) was observed in patients with a premature LH rise (**Table 3**). While in the patients with advanced age, the number of oocytes retrieved and the quality of the embryos were not reduced in the patients with the premature LH rise.

**Table 2:** The pregnancy outcome in cycles with and without a premature LH rise.

	No LH rise	LH rise	*p*
Number of embryos transferred per ET	1.85 ± 0.77	1.78 ± 0.33	0.43
Implantation rate	790/2333 (33.86%)	65/211 (30.81%)	0.37
D3 embryos	121/480 (25.21%)	16/86 (18.60%)	
D5 embryos	669/1853 (36.10%)	49/125 (39.20%)	
Clinical pregnancy rate	577 (61.45%)	47 (57.32%)	0.46
D3 embryos	113 (12.03%)	13 (15.86%)	
D5 embryos	464 (49.41%)	34 (41.46%)	
Ongoing pregnancy rate	493 (52.50%)	42 (51.22%)	0.82
D3 embryos	100 (10.65%)	9 (10.98%)	
D5 embryos	393 (41.85%)	33 (40.24%)	
Abortion rate	93 (16.12%)	7 (14.89%)	0.83
Cumulative live birth rate	484 (51.54%)	40 (49.38%)	0.63
D3 embryos	95 (10.12%)	8 (9.76%)	
D5 embryos	389 (41.43%)	32 (39.02%)	

**Table 3:** Hierarchical analysis of the pregnancy outcomes basing on age between the patients with or without the premature LH rise.

	No of oocytes retrieved		No. of 2PN		No. of available embryos		No. of high-quality embryos		Cumulative live birth rate	
	β (95% CI)	*p*	β (95% CI)	*p*	β (95% CI)	*p*	β (95% CI)	*p*	β (95% CI)	*p*
<37 y	-0.66 (-2.17, 0.85)	0.39	-0.23 (-1.52, 1.06)	0.72	0.07 (-0.25, 0.40)	0.66	-0.08 (-0.37, 0.19)	0.53	1.30 (0.75–2.26)	0.35
≥37 y	-1.10 (-3.04, 0.84)	0.27	-0.59 (-2.27, 1.09)	0.49	0.01 (-0.48, 0.50)	0.96	-0.13 (-0.62, 0.35)	0.58	0.20 (0.05–0.88)	0.03

## Discussion

In this retrospective cohort study, we analyzed the potential effect of a premature rise in LH on the cumulative live birth rate of IVF patients who received GnRH antagonist treatment. This study included 1201 patients and took place over a 3 years period. We demonstrated that in the patients aged ≥37 years-of-age, the occurrence of a premature LH rise was associated with a significantly reduced cumulative live birth rate (β: 0.20; 95% CI: 0.05–0.88; *p* 0.03); this was despite a comparable number of embryos and high-quality embryos.

Gonadotropin-releasing hormone antagonists are widely used in clinics to prevent a premature rise in LH during controlled ovarian stimulation (12–14). However, some patients still experience a premature rise in LH (6); this occurrence has been reported in a range of clinical studies. For example, Kummer et al. (8) and Geng et al. (15) both reported a high incidence of premature LH rises (15.6 and 16.2%, respectively), whereas another study, involving ganirelix, showed a much lower incidence (1.4%) (16). In the present study, 82 patients (8.03%) showed premature LH increases during GnRH antagonist treatment; this finding was in line with those of Dovey et al. (7). These differences may be due to the heterogeneity of the infertile population, the dose of FSH and LH, the time of initiation, and the criteria used to define a premature LH rise.

The effect of a premature LH rise on pregnancy outcome remains controversial. A prospective study of 314 high ovarian responders undergoing a fixed GnRH-antagonist protocol, showed a lower clinical pregnancy rate in patients experiencing a premature rise in LH (15). The same result was found in women undergoing the flexible GnRH antagonist protocol; similarly, reduced rates of ongoing pregnancy rate were also reported previously (7). It has been suggested that the reduced pregnancy outcome may be a reflection of endometrial advancement impairing implantation, as the higher E2 levels associated with higher LH values early in follicular recruitment may induce the expression of progesterone receptors within the endometrium (17). In contrast, other investigations demonstrated that a transient LH rise was not associated with a decline in fertilization, implantation, or pregnancy rates per embryo transfer (8, 18).

However, it should be noted that all the patients in these studies underwent only fresh embryo transfer and did not include frozen embryo transfer. Thus, the effect of a premature rise in LH on the quality of embryos could not be evaluated. The present study provided a unique insight into the effect of a premature rise in LH on the cumulative live birth rate after both fresh and frozen embryo transfer. Our analysis showed that in the patients aged ≥37 years-of-age, the cumulative live birth rate was reduced in the premature LH rise group compared to the patients without the LH premature. This was despite of the fact that the number of embryos acquired, the quality of the embryos in patients of advanced age with or without a premature LH rise were similar. We speculated that embryo potential may be compromised by a premature rise in LH.

Luteinizing hormone (LH) is essential for normal follicular development and oocyte maturation. In a previous study analyzing pregnancy outcome with a fixed *versus* flexible GnRH-antagonist protocol, it was noted that lower LH levels at baseline and the initiation of hCG, were associated with positive pregnancy outcomes (19). Conversely, other studies have indicated that when concentrations of LH were high throughout the follicular phase, the hormone penetrated the follicle and allowed the oocyte to mature prematurely, thus resulting in premature ovulation (20). Meanwhile, other studies have shown that a premature LH rise suppressed the proliferation of granulosa cells and oocytes retrieved from patients with high serum LH were associated with excessive fragmentation and asymmetrical cleavage, thus suggesting that the oocytes were in the early stages of atresia (21, 22). Furthermore, high LH levels during the follicular phase are associated with poor embryo quality and potential (23). In our present study, the cumulative live birth rate was reduced in the premature LH rise group in patients aged ≥37 years-of-age. However, the number of embryos, high-quality embryos and the number of embryo transfer were similar when compared between patients aged ≥37 years with or without a premature rise in LH. Thus, we postulated that the premature rise of LH may affect embryo potential ability in patients of advanced age. However, the reduced cumulative live birth rate was not obvious in our patients who were <37 years-of-age. It is tempting to speculate that the premature rise in LH aggravated the reduced the potential of embryos produced by the advanced age, not the number of embryos.

It should be noted that this is a retrospective study. A prospective study is now crucial if we are to verify the results of the current investigation. Additionally, the progesterone level was not assessed in this study. This was because the aim of the present study was to evaluate the effect of premature rise of LH on the cumulative live birth rate other than the pregnancy outcome in the fresh transfer cycle. But the result will be more persuasive if the progesterone level is considered. Further investigation will focus on the progesterone level in this study.

Overall, our results show that a transient premature rise in LH in a patient undergoing IVF with a GnRH antagonist protocol is associated with a significantly reduced cumulative live birth rate in patients ≥37 years-of-age. This may be due to the detrimental effect of the premature rise in LH on embryonic potential. This may help when counseling a patient as to her chances of future cycle cancellation and in the selection of subsequent treatment protocols.

## Data Availability Statement

The raw data supporting the conclusions of this article will be made available by the authors, without undue reservation.

## Ethics Statement

The study was reviewed and approved by the institutional review board of Peking University Peoples’ Hospital. Written informed consent for participation was not required for this study in accordance with the national legislation and the institutional requirements. Written informed consent was not obtained from the individual(s) for the publication of any potentially identifiable images or data included in this article.

## Author Contributions

HS and HH conceived and designed this study. FG and YW contributed to the acquisition and analyses of data. FG also helped to draft the manuscript. DW, MF, QZ, YR, and ZY were responsible for data collection. HH contributed to revising the manuscript. All authors contributed to the article and approved the submitted version.

## Funding

This study was aided by Project (RD2020-PHB281-01 and RDH2017-04) supported by the Scientific Research Development Fund of Peking University People’s Hospital and by Clinical Medicine Plus X - Young Scholars Project of Peking University, the Fundamental Research Funds for the Central Universities (PKU2021LCXQ020).

## Conflict of Interest

The authors declare that the research was conducted in the absence of any commercial or financial relationships that could be construed as a potential conflict of interest.

## Publisher’s Note

All claims expressed in this article are solely those of the authors and do not necessarily represent those of their affiliated organizations, or those of the publisher, the editors and the reviewers. Any product that may be evaluated in this article, or claim that may be made by its manufacturer, is not guaranteed or endorsed by the publisher.

## References

[1] Huirne JA Lambalk CB. Gonadotropin-Releasing-Hormone-Receptor Antagonists. Lancet (Lond Engl) (2001) 358:1793–803. doi: 10.1016/S0140-6736(01)06797-6 11734258

[2] Copperman AB Benadiva C. Optimal Usage of the GnRH Antagonists: A Review of the Literature. Reprod Biol Endocrinol RB&E (2013) 11:20. doi: 10.1186/1477-7827-11-20 23496864 PMC3618003

[3] Schultze-Mosgau A Griesinger G Altgassen C von Otte S Hornung D Diedrich K. New Developments in the Use of Peptide Gonadotropin-Releasing Hormone Antagonists Versus Agonists. Expert Opin Invest Drugs (2005) 14:1085–97. doi: 10.1517/13543784.14.9.1085 16144493

[4] Stadtmauer LA Sarhan A Duran EH Beydoun H Bocca S Pultz B. The Impact of a Gonadotropin-Releasing Hormone Antagonist on Gonadotropin Ovulation Induction Cycles in Women With Polycystic Ovary Syndrome: A Prospective Randomized Study. Fertil Steril (2011) 95:216–20. doi: 10.1016/j.fertnstert.2010.05.023 20594551

[5] Griesinger G Felberbaum R Diedrich K. GnRH-Antagonists in Reproductive Medicine. Arch Gynecol Obstet (2005) 273:71–8. doi: 10.1007/s00404-005-0021-2 15991015

[6] Sonmezer M Pelin Cil A Atabekoglu C Ozkavukcu S Ozmen B. Does Premature Luteinization or Early Surge of LH Impair Cycle Outcome? Report of Two Successful Outcomes. J Assisted Reprod Genet (2009) 26:159–63. doi: 10.1007/s10815-009-9299-5 PMC2654938 19224360

[7] Dovey S McIntyre K Jacobson D Catov J Wakim A. Is a Premature Rise in Luteinizing Hormone in the Absence of Increased Progesterone Levels Detrimental to Pregnancy Outcome in GnRH Antagonist *In Vitro* Fertilization Cycles. Fertil Steril (2011) 96:585–9. doi: 10.1016/j.fertnstert.2011.06.042 21774931

[8] Kummer NE Weitzman VN Benadiva CA Schmidt DW Engmann LL Nulsen JC. *In Vitro* Fertilization Outcomes in Patients Experiencing a Premature Rise in Luteinizing Hormone During a Gonadotropin-Releasing Hormone Antagonist Cycle. Fertil Steril (2011) 95:2592–4. doi: 10.1016/j.fertnstert.2010.12.046 21292260

[9] Reichman DE Zakarin L Chao K Meyer L Davis OK Rosenwaks Z. Diminished Ovarian Reserve Is the Predominant Risk Factor for Gonadotropin-Releasing Hormone Antagonist Failure Resulting in Breakthrough Luteinizing Hormone Surges in *In Vitro* Fertilization Cycles. Fertil Steril (2014) 102:99–102. doi: 10.1016/j.fertnstert.2014.04.010 24882557

[10] Alpha Scientists in Reproductive Medicine and ESHRE Special Interest Group of Embryology. The Istanbul ConsensusWorkshop on Embryo Assessment: Proceedings of an Expert Meeting. Hum Reprod (2011) 26:1270–83. doi: 10.1093/humrep/der037 21502182

[11] Gardner DK Lane M Stevens J Schlenker T Schoolcraft WB. Blastocyst Score Affects Implantation and Pregnancy Outcome: Towards a Single Blastocyst Transfer. Fertil Steril (2000) 73:1155–8. doi: 10.1016/S0015-0282(00)00518-5 10856474

[12] Lainas T Zorzovilis J Petsas G Stavropoulou G Cazlaris H Daskalaki V. In a Flexible Antagonist Protocol, Earlier, Criteria-Based Initiation of GnRH Antagonist Is Associated With Increased Pregnancy Rates in IVF. Hum Reprod (Oxford England) (2005) 20:2426–33. doi: 10.1093/humrep/dei106 15946995

[13] Lee TH Lin YH Seow KM Hwang JL Tzeng CR Yang YS. Effectiveness of Cetrorelix for the Prevention of Premature Luteinizing Hormone Surge During Controlled Ovarian Stimulation Using Letrozole and Gonadotropins: A Randomized Trial. Fertil Steril (2008) 90:113–20. doi: 10.1016/j.fertnstert.2007.06.029 18054932

[14] Olivennes F Fanchin R Bouchard P de Ziegler D Taieb J Selva J. The Single or Dual Administration of the Gonadotropin-Releasing Hormone Antagonist Cetrorelix in an *In Vitro* Fertilization-Embryo Transfer Program. Fertil Steril (1994) 62:468–76. doi: 10.1016/S0015-0282(16)56933-7 8062940

[15] Geng Y Lai Q Xun Y Jin L. The Effect of Premature Luteinizing Hormone Increases Among High Ovarian Responders Undergoing a Gonadotropin-Releasing Hormone Antagonist Ovarian Stimulation Protocol. Int J gynaecol Obstet: Off Organ Int Fed Gynaecol Obstet (2018) 142:97–103. doi: 10.1002/ijgo.12485 29542120

[16] The Ganirelix Dose-Finding Study Group. A Double-Blind, Randomized, Dose-Finding Study to Assess the Efficacy of the Gonadotrophin-Releasing Hormone Antagonist Ganirelix (Org 37462) to Prevent Premature Luteinizing Hormone Surges in Women Undergoing Ovarian Stimulation With Recombinant Follicle Stimulating Hormone (Puregon). The Ganirelix Dose-Finding Study Group. Hum Reprod (Ox Engl) (1998) 13:3023–31. doi: 10.1093/humrep/13.11.3023 9853849

[17] Kolibianakis E Bourgain C Albano C Osmanagaoglu K Smitz J Van Steirteghem A. Effect of Ovarian Stimulation With Recombinant Follicle-Stimulating Hormone, Gonadotropin Releasing Hormone Antagonists, and Human Chorionic Gonadotropin on Endometrial Maturation on the Day of Oocyte Pick-Up. Fertil Steril (2002) 78:1025–9. doi: 10.1016/S0015-0282(02)03323-X 12413988

[18] Hofmann GE Khoury J Johnson CA Thie J Scott RT Jr. Premature Luteinization During Controlled Ovarian Hyperstimulation for *In Vitro* Fertilization-Embryo Transfer Has No Impact on Pregnancy Outcome. Fertil Steril (1996) 66:980–6. doi: 10.1016/S0015-0282(16)58693-2 8941065

[19] Depalo R Trerotoli P Chincoli A Vacca MP Lamanna G Cicinelli E. Endogenous Luteinizing Hormone Concentration and IVF Outcome During Ovarian Stimulation in Fixed Versus Flexible GnRH Antagonist Protocols: An RCT. Int J Reprod Biomed (Yazd Iran) (2018) 16:175–82. doi: 10.29252/ijrm.16.3.175 PMC5944439 29766148

[20] Homburg R Armar NA Eshel A Adams J Jacobs HS. Influence of Serum Luteinising Hormone Concentrations on Ovulation, Conception, and Early Pregnancy Loss in Polycystic Ovary Syndrome. BMJ (Clinical Res Ed) (1988) 297:1024–6. doi: 10.1136/bmj.297.6655.1024 PMC1834779 3142595

[21] Raju GA Chavan R Deenadayal M Gunasheela D Gutgutia R Haripriya G. Luteinizing Hormone and Follicle Stimulating Hormone Synergy: A Review of Role in Controlled Ovarian Hyper-Stimulation. J Hum Reprod Sci (2013) 6:227–34. doi: 10.4103/0974-1208.126285 PMC3963304 24672160

[22] Shoham Z. The Clinical Therapeutic Window for Luteinizing Hormone in Controlled Ovarian Stimulation. Fertil Steril (2002) 77:1170–7. doi: 10.1016/S0015-0282(02)03157-6 12057724

[23] Kolibianakis EM Albano C Kahn J Camus M Tournaye H Van Steirteghem AC. Exposure to High Levels of Luteinizing Hormone and Estradiol in the Early Follicular Phase of Gonadotropin-Releasing Hormone Antagonist Cycles Is Associated With a Reduced Chance of Pregnancy. Fertil Steril (2003) 79:873–80. doi: 10.1016/S0015-0282(02)04920-8 12749423

